# Retrospective Application of Risk Scores to Unruptured Anterior Communicating Artery Aneurysms

**DOI:** 10.3390/jcm13030789

**Published:** 2024-01-30

**Authors:** Katarzyna Wójtowicz, Lukasz Przepiorka, Sławomir Kujawski, Edyta Maj, Andrzej Marchel, Przemysław Kunert

**Affiliations:** 1 Department of Neurosurgery, Medical University of Warsaw, 02-097 Warsaw, Polandamarchel@wum.edu.pl (A.M.); pkunert@wp.pl (P.K.); 2 Department of Exercise Physiology and Functional Anatomy, Ludwik Rydygier Collegium Medicum in Bydgoszcz, Nicolaus Copernicus University in Toruń, 85-077 Bydgoszcz, Poland; 3 Second Department of Clinical Radiology, Medical University of Warsaw, 02-097 Warsaw, Poland; em26@wp.pl

**Keywords:** unruptured intracranial aneurysm, anterior communicating artery, UIATS, PHASES, concordance

## Abstract

Background: Treatment decisions for unruptured intracranial aneurysms (UIAs) pose a challenge for neurosurgeons, prompting the development of clinical scales assessing hemorrhage risk to provide management guidance. This study compares recommendations from the PHASES and UIA treatment scores (UIATS) applied to anterior communicating artery (AComA) UIAs against real-world management. Methods: While UIATS recommends management, for PHASES, an aneurysm with score of 10 or more was considered “high-risk”. Analysis involved assessing the concordance in each group alongside comparison to real-word management. Results: Among 129 patients, 46.5% were observed and 53.5% were treated. PHASES scores were significantly higher in the treatment group (*p* = 0.00002), and UIATS recommendations correlated with real-world decisions (*p* < 0.001). We observed no difference in the frequencies of UIATS recommendations between high- and low-risk groups. When comparing the UIATS and PHASES, 33% of high-risk aneurysms received a UIATS conservative management recommendation. In 39% of high-risk aneurysms, the UIATS recommendation was not definitive. Conversely, 27% of low-risk aneurysms obtained a UIATS UIA repair recommendation. Overall, concordance between PHASES and UIATS was 32%. Conclusions: Significant discordance in therapeutic suggestions underscores the predominant influence of center experience and individual assessments. Future studies should refine and validate decision-making strategies, potentially exploring alternative applications or developing tailored scales.

## 1. Introduction

With the heightened availability and improved precision of diagnostic imaging, in recent years there has been a notable surge in the detection rate of asymptomatic unruptured intracranial aneurysms (UIAs), particularly among elderly patients [1]. This trend is accompanied by a noteworthy inclination to qualify smaller aneurysms for treatment in older patient cohorts. Presumably, this shift in clinical practice is influenced by the advancing safety profile of aneurysm treatment modalities [2,3].

Nevertheless, navigating the decision of whether to treat a UIA presents a challenging dilemma for neurosurgeons worldwide, as well as remaining an ever-present concern in their daily practice. Consequently, several scales have been developed to assess the risk of hemorrhage and offer treatment recommendations. A number of studies have analyzed the usefulness of these scales in predicting the risk of hemorrhage in populations with ruptured intracranial aneurysms, and they show that a large percentage of ruptured aneurysms would have been assigned as low risk aneurysms pre-rupture [4,5,6,7].

In this evolving landscape of UIA management, anterior communicating artery (AComA) UIAs stand out. Their distinctive features include high prevalence, proximity to functionally vital structures, and a speculated increased risk of rupture. The aim of this study is to compare management recommendations for AComA UIAs specifically, evaluate different protocols, and compare them with each other and to real-life management of AComA UIAs in a single institution.

## 2. Materials and Methods

This study is a retrospective evaluation of patients with AComA UIAs who were observed or treated. These patients, including their follow-ups and complications, have already been extensively described in our previous study [8]. In this paper, we applied PHASES and UIATS in comparison to real-life management at a single institution [9,10].

We analyzed the concordance of these scales in each group and compared them to our management. We analyzed a variety of demographics and aneurysm factors to find the differences between the subgroups of different recommendations. The recommendations from UIATS are categorized as either definitive (‘UIA repair’, i.e., treatment or ‘conservative management’, i.e., observation) or non-definitive (‘the recommendation is not definitive’).

This paper will interchangeably refer to recommendations derived from UIA clinical scales and real-world management. To ensure clarity throughout the text, specific terms will be introduced and utilized consistently. We will refer to the real-world aneurysm cohorts as belonging to either the ‘treatment’ or ‘observation’ group. Regarding UIATS recommendations, the terms ‘UIA repair’ or ‘conservative management’ will be used, as described in the original paper. For the PHASES scale, which assesses the 5-year hemorrhage risk, a score of 10 or more assigned to the aneurysm was considered high-risk, as proposed by Stumpo et al. [11]. Accordingly, whenever the terms ‘high-risk’ or ‘low-risk’ aneurysm are utilized in this paper, they will correspond to the PHASES score interpretation.

### Statistical Analysis

We performed the Shapiro-Wilk and Levene’s tests to examine the assumptions of data normality and the equality of variances, respectively. We examined the associations between qualitative variables with the Chi-squared and Fisher’s exact tests. The Chi-square Goodness of Fit test was used to summarize the discrepancy between observed values and expected values. Differences between the two groups were assessed using either the Mann-Whitney U test or independent *t*-tests, depending on whether assumptions were met. We reported *p*-values both before and after false discovery rate (FDR) correction. Differences between more than two groups were evaluated with the Kruskal-Wallis H test or one-way ANOVA, depending on whether assumptions were met. Post-hoc comparisons were made using the Dunn test, and *p*-values were adjusted for multiple comparisons. All analyses were performed using jamovi, which is a graphical user interface for R and violin graphs, which were created using the ggstatsplot library [12,13,14]. Effect size (ε) and confidence interval [−95%; 95%] from the ggstatsplot library are reported for Kruskal-Wallis test results. All analyses were performed with a significance level of α = 0.05.

To mitigate potential high correlation between PHASES and age, as well as UIATS and age, we avoided including these variables as predictors in the same model. Instead, we independently considered age, PHASES, and UIATS in separate models, allowing a distinct assessment of their contributions to our analyses.

## 3. Results

In our series of 129 patients with AComA UIAs, 46.5% (60) of patients remained under observation and 53.5% (69) were qualified for treatment.

### 3.1. PHASES Scores and Real-World Management Decisions

There were 111 (86%) AComA aneurysms classified as low-risk (score < 10) and 18 (14%) classified as high-risk (score ≥ 10), according to the PHASES scores.

**Figure 1:**
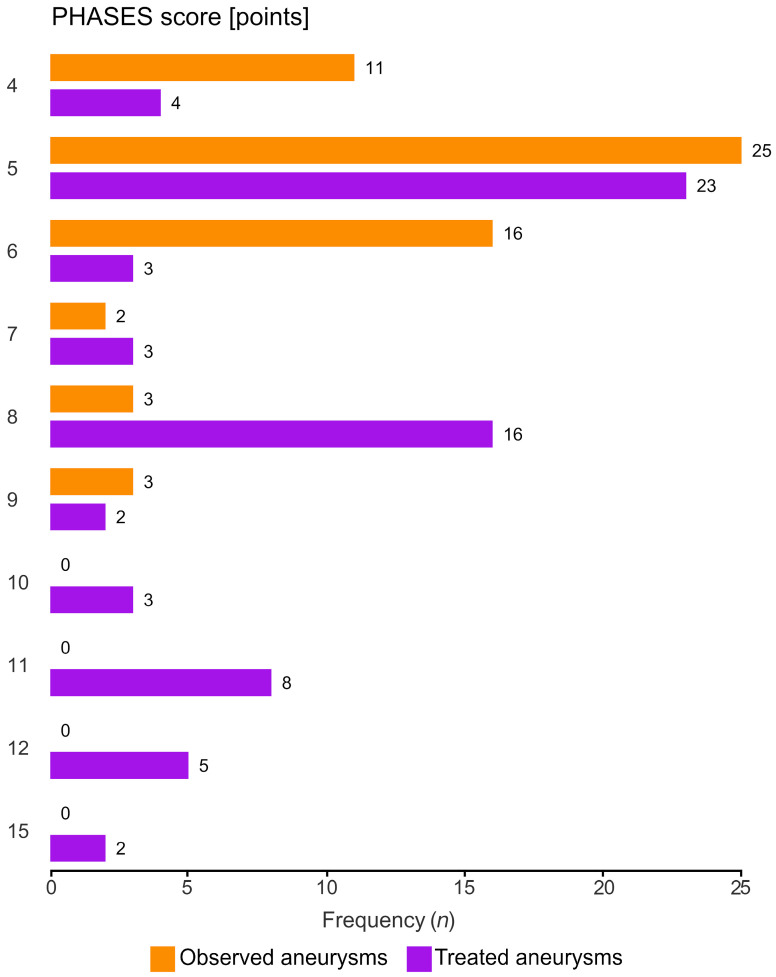
Distribution of PHASES scores across treated and observed unruptured anterior communicating artery aneurysms. Orange—observed aneurysms, violet—treated aneurysms.

The PHASES scores were significantly higher in the group qualified for treatment [median ± interquartile range (IQR) was 8 ± 5 in the treatment group vs. 5 ± 1 in the observation group, *p* = 0.00002, Table 1]. Figure 1 presents the distribution of treated and observed AComA aneurysms according to their PHASES scores.

### 3.2. UIATS and Real-World Management Decisions

In accordance with UIATS, UIA repair was recommended for 27% (35) of patients and conservative management for 32% (41) of patients, while the recommendation remained not definitive for 37% (48) of individuals. In five cases, our data was insufficient to calculate UIATS. Patients under observation more frequently received an UIATS conservative management recommendation and, correspondingly, patients who qualified for treatment more frequently received a UIATS UIA repair recommendation (Chi-square test, *p* < 0.001, Table 2). A significant association was identified between UIATS recommendations for UIA repair or conservative management and real-world decisions (Fisher’s exact test, *p* < 0.001).

### 3.3. UIATS Recommendations against PHASES Scores

We observed no difference in the frequencies of UIATS recommendations between high and low-risk groups, as interpreted by PHASES (Table 3, for details please refer to Supplementary Table S1).

**Figure 2:**
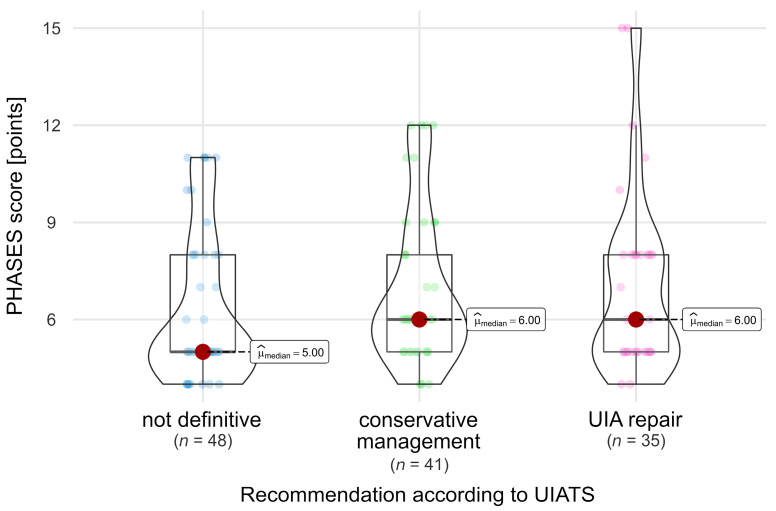
Comparison of PHASES scores in relation to the UIATS recommendation across the whole cohort. A dark red dot denotes the median value, while smaller dots indicate values of individual patients.

In the entire cohort, PHASES scores were not significantly different in relation to any UIATS recommendation (Chi-squared Kruskal-Wallis = 4.91, *p* = 0.086, Figure 2).

In the subgroup of aneurysms with a definitive recommendation, UIATS recommended conservative management for 55% (6) of high-risk and for 54% (35) of low-risk aneurysms, according to PHASES score interpretation. Likewise, in the subgroup with a UIATS definitive recommendation, UIA repair was recommended for 46% (30) of low-risk and for 45% (5) of high-risk aneurysms, as per PHASES score interpretation (chi-squared *p*-value 0.97). 

### 3.4. Observation Group

PHASES scores in the group of 60 patients under observation ranged from 4 to 9, and no high-risk aneurysms (according to PHASES interpretation) were identified. In this group, UIATS recommended UIA repair for 11% (6) of patients and conservative management for 51% (28) of patients, while 38% (21) of patients lacked a specific recommendation (i.e., the UIATS recommendation was not definitive).

**Figure 3:**
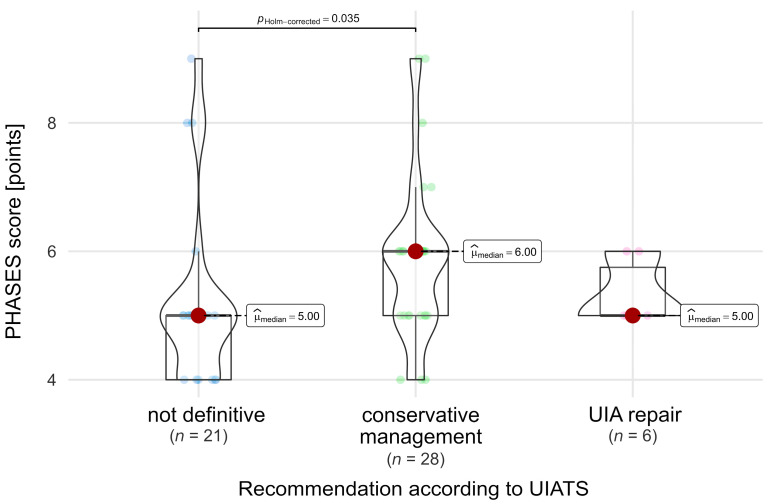
Comparison of PHASES scores in relation to UIATS recommendations in the observation group. A dark red dot denotes the median value, while smaller dots indicate values of individual patients.

Among patients under observation, PHASES scores were significantly different between subgroups based on UIATS recommendation [Chi-squared Kruskal-Wallis = 6.35, *p* = 0.042, ε2 = 0.12 (0.02; 1)]. The subgroup with a UIATS conservative management recommendation had significantly higher PHASES scores when compared against patients with ‘not definitive’ recommendations (*p* = 0.035, Figure 3).

We compared patients whom we observed following UIATS recommendations for conservative management against those whom we observed despite UIATS recommending UIA repair. Among the patients under observation in real-life management, we identified a subgroup with a UIATS UIA repair recommendation, displaying distinctive characteristics. Specifically, these patients were significantly younger [median (IQR) age of 51 (48 to 57) years old vs. median (IQR) of 72 (68 to 77) years old], had more frequent previous SAH (*p* = 0.025), a family history of aneurysms and/or a hemorrhage from another aneurysm in the past (*p* = 0.016), were more often active smokers (*p* = 0.016), and had multiple aneurysms significantly more often (*p* = 0.021, Table 4).

### 3.5. Treatment Group

**Figure 4:**
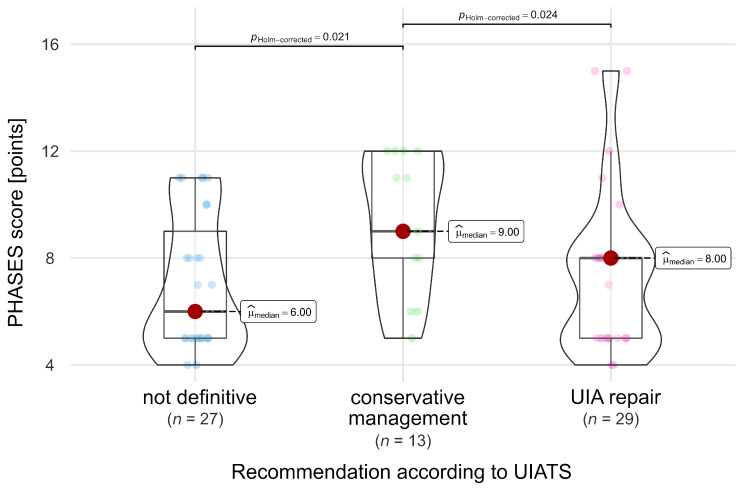
Comparison of PHASES scores in relation to UIATS recommendations in patients who underwent treatment. A dark red dot denotes the median value, while small dots indicate values of individual patients.

PHASES scores in the group of patients who underwent treatment ranged from 4 to 15, with 26% (18) of them having high-risk aneurysms (PHASES score of 10 or more). According to UIATS, UIA repair was recommended for 42% (29) of patients and conservative management for 19% (13) of patients, and a recommendation was not definitive for 39% (27). The PHASES scores in these three subgroups of different UIATS recommendations among patients who underwent treatment were significantly different [Chi-squared Kruskal-Wallis = 8.12, *p* = 0.017, ε2 = 0.12 (0.04; 1); Figure 4]. Notably, within the treatment group, patients with UIATS conservative management recommendations had significantly higher PHASES scores compared to other treated patients, namely those for whom UIATS recommended UIA repair or did not provide a clear recommendation (‘recommendation not definitive’).

We additionally compared the aneurysms within our treatment group, distinguishing between those for which UIATS recommended UIA repair and those for which UIATS recommended conservative management. Patients with UIATS UIA repair recommendation (within the real-world treatment group) were significantly younger [median (IQR) age of 54 (48 to 58) years old vs. median (IQR) of 71 (67 to 75) years old, *p* < 0.001)], were active smokers more frequently (69% vs. 0%, *p* < 0.001), but had lower PHASES scores [median (IQR) of 8 (5 to 8) vs. median (IQR) of 9 (8 to 12), *p* = 0.007], when compared against the patients within the treatment group who received UIATS observation recommendation (Table 5).

### 3.6. Summary of the Results

As a result of our management strategy, we reserved observation only for low-risk AComA UIAs according to the PHASES score interpretation. Even so, concurrently, we treated as many as 46% (51) of low-risk aneurysms. Overall, our management was in concordance with the PHASES score interpretation in 60% of cases.

In 60% (76) of cases, UIATS recommendations were definitive, while the remaining cases were categorized as not definitive. We treated 82% (29) of cases in which UIATS recommended UIA repair and observed 68% (28) of cases for which UIATS recommended conservative management. Overall, our management was in concordance with UIATS recommendations in 46% of cases.

Surprisingly, the concordance of these two scales applied to AComA UIAs was as low as 32% (40). When comparing the UIATS and PHASES evaluations, we found that 33% (6 cases) of high-risk aneurysms (as per PHASES interpretation) received a UIATS conservative management recommendation. In as many as 39% (7) of these high-risk aneurysms, the UIATS recommendation was not definitive. On the other hand, 27% (30) of cases of low-risk aneurysms obtained a UIATS UIA repair recommendation.

In our analysis of AComA UIAs that underwent treatment, an intriguing pattern emerged when evaluating the recommendations from UIATS and PHASES. Notably, a mere 7.2% of treated AComA UIAs received a UIATS UIA repair recommendation while simultaneously being deemed high-risk according to their PHASES interpretation. The majority of treated aneurysms (61%) fell into either the high-risk category based on their PHASES interpretation, or received a UIA repair recommendation from UIATS.

## 4. Discussion

The AComA complex is a prevalent site for UIAs, constituting up to 33% of cases. A number of studies underscore its elevated risk of rupture, as indicated by a meta-analysis reporting a 2.51 times higher risk for AComA aneurysms compared to those in other anterior circulation sites [15,16]. Moreover, small AComA UIAs have been reported to bear a similar risk of rupture to posterior circulation aneurysms [17]. The conjunction and synergy of these two features established the foundation for distinguishing AComA aneurysms from other locations within the anterior circulation in our studies.

The PHASES score emerged from a collaborative effort among researchers from diverse institutions, originating in a large international study that synthesized data from individual patient records and aneurysm databases [9]. Developed by analyzing various risk factors and their associations with aneurysm rupture, the PHASES score serves as a tool for clinicians to estimate rupture risk in UIAs based on individual patient characteristics [18].

The UIATS recommendations, representing a consensus effort among researchers, were developed through a comprehensive analysis of data from various sources, including individual patient records and aneurysm databases [10]. These guidelines provide clinicians with evidence-based insights for managing UIAs [19].

### 4.1. UIATS Recommendations

In our investigation, we identified instances where the recommendations provided by UIATS were not clearly delineated. This raises concerns about the suitability of using UIATS in isolation. Notably, patients who were observed in real-life settings more frequently received a UIATS recommendation for conservative management than a recommendation for UIA repair. Nevertheless, 11% of these patients (6) were recommended to undergo UIA repair. This subset of patients, receiving a UIATS UIA repair recommendation and who remained under observation in real life, exhibited distinct characteristics—they were notably younger, had a higher incidence of familial history related to aneurysms or prior hemorrhages from another aneurysm, were more likely to smoke, and presented with multiple aneurysms. Interestingly, the aneurysms in the observation group with a UIATS UIA repair recommendation were very small (median size 2.1 mm), even when compared to those in the subgroup receiving a UIATS conservative management recommendation, although this difference was not statistically significant. This persistent UIA repair recommendation was noteworthy, particularly in light of three points favoring conservative management, which were justified by the aneurysm’s complexity arising from a very small diameter (<3 mm).

It is worth noting that, within the treatment group, significantly older patients consistently received UIATS conservative management recommendations. This feature of UIATS, evident in patients who underwent treatment in our real-life scenario, harmonizes with the study by Rutledge et al. [20]. The researchers reported that UIATS tends to underestimate the risk of hemorrhage in older patients. Moreover, older patients are more severely affected by aneurysmal hemorrhage [21,22]. This emphasizes the need for a critical evaluation of UIATS recommendations in older populations, recognizing the potential underestimation of hemorrhage risk and the heightened severity of aneurysmal hemorrhage in this demographic.

### 4.2. PHASES Scores

Although patients who underwent preventive aneurysm repair had significantly higher PHASES scores, we treated almost half (46%, 51) of patients with low–risk aneurysms (according to PHASES interpretation). An additional analysis of these cases unveiled that the vast majority (96%, 49) harbored at least one type A risk factor, and two-thirds (66.7%, 34) had two type A risk factors, according to Chalouhi et al. [23]. The remaining 4% of patients had one type A risk factor and one type B factor, which advocates for treatment, according to the researcher’s algorithm. In our management approach, we did not observe any high-risk aneurysms (as per PHASES interpretation).

### 4.3. UIATS and PHASES Comparison

As presented in the summary of the results, a notable discordance between PHASES and UIATS persists in AComA UIAs: every third high-risk aneurysm, as per PHASES interpretation, received a UIA repair recommendation according to UIATS. Overall, the concordance of these scales was 32%. It would be reasonable to assume that using more tools should help with daily clinical practice, but our results show that the use of these scales did not make decision-making simpler, instead adding to the confusion on what to do. In certain scenarios, it appears likely that UIATS recommends conservative management for high-risk aneurysms (as per PHASES) due to the consideration of treatment-related risks. Unfortunately, an analogous explanation for why UIATS recommends UIA repair for low-risk aneurysms in certain cases remains elusive.

What is more, we observed two unusual findings - one within the observation group, and another within the treatment group. Firstly, among patients under observation, the subgroup with a UIATS conservative management recommendation had significantly higher PHASES scores compared to patients with a “not definitive” recommendation. The apparent contradiction in this finding may be attributed to the objective of UIATS construction, which considers not only aneurysm characteristics but also the inherent risks of treatment. Such a comprehensive approach may lead to a final recommendation favoring conservative management, despite apparent higher aneurysm risks depicted by relatively higher PHASES scores.

Secondly, within the treatment group, patients with UIATS conservative management recommendations had significantly higher PHASES scores compared to other treated patients, specifically those for whom UIATS recommended UIA repair or did not provide a clear recommendation. This apparent paradox may be simply explained by our management approach, wherein patients with high-risk aneurysms (as per PHASES interpretation) were qualified for treatment, regardless of other associated risks indicated in the UIATS recommendations. In essence, our decision to opt for patient treatment was influenced by the recommendation of only one of the scales.

### 4.4. Limitations of Aneurysm Scale Use

Scales intended to forecast the risk of aneurysm hemorrhage or offer clinical guidance are not commonly integrated into clinical practice [24]. Additionally, these scales suffer from a notable deficit of prospective evaluation, a crucial element requisite for establishing a robust scientific basis to support their widespread implementation [19]. On the contrary, there are a number of studies that have been critical of using these scales. Ravindra et al. concluded that UIATS recommended the overtreatment of unruptured aneurysms [19]. When analyzing ruptured aneurysms, Rutledge et al. found that applying UIATS to elderly patients would have led to their undertreatment, a problem not observed in younger populations [20]. Furthermore, Hernandez-Duran et al. found that the sensitivity of UIATS in detecting high-risk aneurysms in ruptured aneurysm cases was low [25]. On the contrary, Feghali et al. showed that UIATS demonstrated good concordance with real-world practice [26].

A major shortcoming of UIATS was recently reported by Stumpo et al. [5]. The researchers claimed that UIATS would have failed to recommend UIA repair in 72.6% of patients whose aneurysms eventually ruptured. Additionally, Molenberg et al. reported poor performance of UIATS in predicting aneurysm growth or rupture [27].

### 4.5. Study Limitations

This study possesses inherent limitations attributed to its retrospective design, a relatively modest sample size, and single-center focus. While the latter introduces a degree of subjectivity to the patient group, management processes, and results evaluation, we contend that this limitation offers unique strengths within the specific context of our investigation.

The homogeneity observed in the evaluated AComA UIAs, coupled with the consistent approach to management decisions (albeit with some adjustments over time) resulting from our single-center methodology, provides a distinctive advantage. This homogeneity facilitates direct comparisons, enabling the derivation of meaningful conclusions—a feat that might prove challenging in a multicenter, retrospective study design with potentially increased variability in patient populations and management practices. The inclusion of numerous centers may introduce additional confounding factors, complicating the interpretation of results.

Furthermore, as previously noted in our earlier publication, a subset of patients from external medical facilities qualified for observation. However, their data are not incorporated into our databases [8]. Consequently, the reported number of patients under observation in this study represents only a fraction of the total AComA UIAs under observation, excluding those managed at external hospitals and outpatient clinics. This omission emphasizes the necessity for cautious generalization of our findings to broader patient populations.

Finally, an important limitation of our study lies in the use of the PHASES score interpretation. Following the approach outlined by Stumpo et al., we categorized aneurysms into high- and low-risk groups, subsequently assigning them to treatment and observation, respectively [11]. It is crucial to acknowledge this methodology when interpreting the results of our investigation. However, the necessity for some degree of generalization, including the use of, at times, arbitrary cutoffs, is recognized to facilitate evaluation in a broader context.

### 4.6. Final Remarks and Future Directions

Assessing the concordance of aneurysm management with PHASES scores proves challenging, as PHASES scoring primarily estimates the risk of hemorrhage rather than guiding aneurysm management. As per the European Stroke Organisation guidelines, preventive aneurysm repair is recommended for individuals with a 5-year risk of aneurysm rupture, as this surpasses the risks associated with preventive treatment [28]. The impact of implementation of the PHASES score in aneurysm management was evaluated by Hollands et al. [29]. The researchers found that out of two examined centers, one did not change its previous practice, while the other began to qualify less aneurysms for treatment.

In our management, we disqualified high-risk aneurysms from observation, and a substantial number of low-risk aneurysms were treated (as assessed with PHASES interpretation). This finding aligns with the observations of Longnon et al., who reported that PHASES did not identify the majority of patients as being at a high or intermediate risk of rupture [6]. Hilditch et al. reported comparable results [30].

The second noteworthy finding is the divergence in suggestions between PHASES and UIATS in numerous cases. While this initial discrepancy may complicate decision-making, a potential strategy could involve using the PHASES scale to estimate the risk of hemorrhage. Subsequently, if an aneurysm is identified as high risk, the UIATS scale could serve as a more refined tool to strike a balance between the risk of rupture and the risk of treatment. Such a scheme necessitates future prospective evaluation.

Furthermore, prospective multicenter studies, utilizing established frameworks like UIATS and PHASES, are crucial and much needed for advancing our understanding of AComA UIAs. A collaborative, prospective approach in properly designed multicenter studies would enhance the generalizability of findings, capturing diverse patient demographics and management practices. These studies should focus on elucidating natural history and risk factors of UIAs, as well as establishing a consensus on optimal management and the usefulness of clinical scales. Moreover, crucial additional outcomes to consider encompass the real-world morbidity associated with interventions for UIAs and their consequential impact on patients’ quality of life. The use of standardized assessment tools ensures a consistent framework, contributing to evidence-based guidelines for improved clinical decision-making.

## 5. Conclusions

Over two thirds of the evaluations in the AComA UIA group yielded discordant suggestions between PHASES and UIATS assessments. This underscores that therapeutic decisions continue to be primarily influenced by the center’s experience, individual assessments, and patient preferences. Recognizing the shortcomings of currently used scales, future prospective studies are needed to refine and validate decision-making strategies for managing UIAs. This may involve exploring alternative applications of current scales and developing new ones tailored to specific patients and aneurysms.

## Figures and Tables

**Table 1:** PHASES scores in patients with unruptured anterior communicating artery aneurysms who were under observation or were qualified for treatment.

Group	*n*	PHASES Scores	
		Median	Range
Treatment	69	8	4–15
Observation	60	5	4–9

**Table 2:** UIATS recommendations for patients with unruptured anterior communicating artery aneurysms who were under observation or qualified for treatment. UIA—unruptured intracranial aneurysm.

Group	Recommendation According to UIATS			No Data
	UIA Repair	Conservative Management	Not Definitive	
Observation, *n* = 60	6 (10%)	28 (47%)	21 (35%)	5 (8%)
Treatment, *n* = 69	29 (42%)	13 (19%)	27 (39%)	0

**Table 3:** A comparative analysis of aneurysm evaluations according to PHASES and UIATS recommendations. Aneurysms with PHASES score of 10 and more were classified as high-risk. UIA—unruptured intracranial aneurysm.

PHASES Interpretation of an Aneurysm	Recommendation According to UIATS			Chi-Square Goodness of Fit *p*-Value
	UIA Repair	Conservative Management	Not Definitive	
Low-risk	30 (28%)	35 (33%)	41 (39%)	0.424
High-risk	5 (28%)	6 (33%)	7 (39%)	0.846

**Table 4:** Demographic and aneurysmal information for UIATS recommending unruptured intracranial aneurysm (UIA) repair or conservative management within a group of patients with unruptured anterior communicating artery aneurysms under observation. FDR—false discovery rate, IQR—interquartile range, NA—not applicable, SAH—subarachnoid hemorrhage, UIA—unruptured intracranial aneurysm.

Parameter (Unit)	Recommendation According to UIATS		*p*-Value	*p*-Value FDR
	Conservative Management [*n* (%)/Median (IQR)]	UIA Repair [*n* (%)/Median (IQR)]		
Patients	28	6		
Age (years)	72 (68 to 77)	51 (48 to 57)	<0.001	0.008
Aneurysm size (mm)	3.94 (2.28 to 6.00)	2.10 (1.55 to 4.00)	0.094	0.12
Previous SAH for another aneurysm	1 (3.6%)	3 (50%)	0.012	0.022
Familial history	0	3 (50%)	0.003	0.014
Active smoking	5 (18%)	5 (83%)	0.005	0.014
Multiple aneurysms	6 (21%)	5 (83%)	0.008	0.019
Aspect ratio > 1.6	1.25 (0.97 to 1.75)	1.27 (0.67 to 1.60)	0.60	0.67
Size ratio > 3	1.88 (1.25 to 2.64)	0.99 (0.78 to 1.77)	0.040	0.06
Low-risk aneurysm *	28	6	NA	NA
High-risk aneurysm *	0	0		

* Evaluated with PHASES interpretation, in which a score of 10 or more assigned to the aneurysm was considered high-risk, while scores under 10 were low-risk.

**Table 5:** Demographic and aneurysmal information for UIATS recommending UIA repair or conservative management within groups of patients with unruptured anterior communicating artery aneurysms who underwent treatment. FDR—false discovery rate, IQR—interquartile range, SAH—subarachnoid hemorrhage, UIA—unruptured intracranial aneurysm.

Parameter (Unit)	Recommendation According to UIATS		*p*-Value	*p*-Value FDR
	Conservative Management [*n* (%)/Median (IQR)]	UIA Repair [*n* (%)/Median (IQR)]		
Patients	13	29		
Age (years)	71 (67 to 75)	54 (48 to 58)	<0.001	<0.001
Aneurysm size (mm)	8.2 (7.5 to 10.2)	7.0 (5.5 to 9.0)	0.10	0.16
Previous SAH from another aneurysm	0	2 (6.9%)	>0.99	>0.99
Familial history	0	7 (24%)	0.079	0.16
Active smoking	0	20 (69%)	<0.001	<0.001
Multiple aneurysms	2 (15%)	13 (45%)	0.089	0.16
Multilobulated aneurysms	4 (31%)	9 (31%)	>0.99	>0.99
Aspect ratio > 1.6	1.79 (1.39 to 2.24)	1.56 (1.30 to 2.19)	0.97	>0.99
Size ratio > 3	4.32 (2.82 to 5.26)	3.12 (2.69 to 3.89)	0.059	0.16
Low-risk aneurysm *	7	23	0.16	0.22
High-risk aneurysm *	4	3		

* Evaluated with PHASES interpretation, in which a score of 10 or more assigned to the aneurysm was considered high-risk, while scores under 10 were low-risk.

## Data Availability

The data presented in this study are available on request from the corresponding author after acceptance of all the co-authors.

## Supplementary Materials

The following supporting information can be downloaded at: https://www.mdpi.com/article/10.3390/jcm13030789/s1, Supplementary Table S1. Distribution of the PHASES scores across UIATS recommendations in patients with unruptured anterior communicating artery aneurysms. UIA—unruptured intracranial aneurysm.
