# Supplementary material for: Retrospective Application of Risk Scores to Unruptured Anterior Communicating Artery Aneurysms
Source: J Clin Med. 2024 Jan 30;13(3):789. doi: 10.3390/jcm13030789 (PMC10856240; doi:10.3390/jcm13030789)
Supplement: Supplementary file 1 [file jcm-13-00789-s001.zip › jcm-2818191-supplementary.pdf]

PHASES score	Recommendation according to UIATS			Sum
	UIA repair	conservative management	not definitive	
4	2 (15%)	3 (23%)	8 (62%)	13 (100%)
5	15 (33%)	9 (19%)	22 (48%)	46 (100%)
6	2 (11%)	14 (78%)	2 (11%)	18 (100%)
7	1 (20%)	2 (40%)	2 (40%)	5 (100%)
8	10 (53%)	3 (11%)	6 (31%)	19 (100%)
9	0	4 (80%)	1 (20%)	5 (100%)
10	1 (33%)	0	2 (67%)	3 (100%)
11	1 (12%)	2 (25%)	5 (63%)	8 (100%)
12	1 (20%)	4 (80%)	0	5 (100%)
15	2 (100%)	0	0	2 (100%)

Supplementary Table S1. Distribution of the PHASES scores across UIATS recommendations in patients with unruptured anterior communicating artery aneurysms. UIA — unruptured intracranial aneurysm
